# Association of Sodium-Glucose Cotransporter 2 (SGLT2) Inhibitor Use With Cardiovascular and Renal Outcomes in Type 2 Diabetes Mellitus Patients With Stabilized Acute Myocardial Infarction: A Propensity Score Matching Study

**DOI:** 10.3389/fcvm.2022.882181

**Published:** 2022-04-29

**Authors:** Ting-Yung Chang, Chi-Ting Lu, Hsin-Lei Huang, Ruey-Hsing Chou, Chun-Chin Chang, Chung-Te Liu, Po-Hsun Huang, Shing-Jong Lin

**Affiliations:** ^1^Cardiovascular Research Center, National Yang Ming Chiao Tung University, Taipei, Taiwan; ^2^Division of Cardiology, Department of Medicine, Taipei Veterans General Hospital, Taipei, Taiwan; ^3^Department of Nursing, National Taipei University of Nursing and Health Sciences, Taipei, Taiwan; ^4^Department of Critical Care Medicine, Taipei Veterans General Hospital, Taipei, Taiwan; ^5^Graduate Institute of Clinical Medicine, College of Medicine, Taipei Medical University, Taipei, Taiwan; ^6^Division of Nephrology, Department of Internal Medicine, Wan-Fang Hospital, Taipei Medical University, Taipei, Taiwan; ^7^Taipei Heart Institute, Taipei Medical University, Taipei, Taiwan; ^8^Heart Center, Cheng-Hsin General Hospital, Taipei, Taiwan

**Keywords:** diabetes, mortality, hospitalization, SGLT2 inhibitor, myocardial infarction

## Abstract

**Background:**

Coronary artery disease (CAD) is one of the leading causes of morbidity and mortality in patients with type 2 diabetes mellitus (T2DM), who are at a greater risk of acute myocardial infarction (AMI) and sudden cardiac death. Sodium-glucose cotransporter 2 (SGLT2) inhibitors have been shown to reduce cardiovascular events and mortality in T2DM patients with a risk of cardiovascular disease. This study aimed to investigate the effect of SGLT2 inhibitor use on the adverse cardiovascular and renal outcomes in T2DM patients with AMI.

**Methods:**

A total of 1,268 patients admitted to the Coronary Care Unit due to AMI were retrospectively screened.Patients taking SGLT2 inhibitors before or during the index AMI hospitalization were assigned as group 1. Patients who never received SGLT2 inhibitors were assigned as group 2. Patients in groups 1 and 2 were matched in a 1:2 ratio, and 198 T2DM patients with stabilized AMI were retrospectively enrolled for the final analysis.

**Results:**

With a mean follow-up period of 23.5 ± 15.7 months, 3 (4.5%) patients in group 1 and 22 (16.7%) patients in group 2 experienced rehospitalization for acute coronary syndrome (ACS), while 1 (1.5%) patient in group 1 and 7 (5.3%) patients in group 2 suffered sudden cardiac death. The Kaplan–Meier curves demonstrated that the patients in group 1 had a lower risk of adverse cardiovascular outcomes. According to the multivariate analysis, the baseline estimated glomerular filtration rate (eGFR) (*P* = 0.008, 95% CI: 0.944–0.991) and the use of SGLT2 inhibitors (*P* = 0.039, 95% CI: 0.116–0.947) were both independent predictors of adverse cardiovascular outcomes. On the other hand, the use of SGLT2 inhibitors was not associated with adverse renal outcomes.

**Conclusion:**

In T2DM patients with stabilized AMI, the use of SGLT2 inhibitors was associated with a lower risk of adverse cardiovascular outcomes. In addition, the baseline renal function was also an independent predictor of adverse cardiovascular outcomes.

## Introduction

Coronary artery disease (CAD) is one of the leading causes of morbidity and mortality in patients with type 2 diabetes mellitus (T2DM) (1–3). Patients with T2DM are at a greater risk of acute myocardial infarction (AMI), heart failure, and sudden cardiac death (4–7). A report by the Global Registry of Acute Coronary Events has demonstrated that in CAD patients presented to hospitals, approximately 1 out of 4 has a history of T2DM, which shows the high co-occurrence rate of T2DM and CAD (8). In addition, previous studies have revealed that T2DM patients and CAD patients have a similar risk for sudden cardiac death and major adverse cardiovascular events (MACE) (9). Therefore, these patients are considered as a single population regarding their risk for sudden cardiac death and MACE (10, 11).

Sodium-glucose cotransporter (SGLT) 2 inhibitors comprise a novel class of oral hypoglycemic agents that has been shown to improve cardiovascular outcomes in patients with T2DM and heart failure (11–14). Recently, four cardiovascular outcome trials (CVOTs) have been conducted to explore the safety and efficacy of SGLT2 inhibitors on cardiovascular outcomes in T2DM patients with a high risk of cardiovascular events (EMPA-REG OUTCOME, DECLARE-TIMI 58, CANVAS, and VERTIS-CV) (15–18). Based on the data of these CVOTs, a meta-analysis concluded that SGLT2 inhibitors reduce the risk for cardiovascular events and mortality, especially in patients with both T2DM and CAD at baseline (19).

Based on these results, the 2019 European Society of Cardiology Guidelines on Diabetes and Cardiovascular Diseases listed SGLT2 inhibitors as one of the first-line glucose-lowering drugs for the treatment of T2DM patients with a high risk of cardiovascular disease (20). Nevertheless, although previous studies have shown the effect of SGLT2 inhibitors on diabetic patients at a high risk for cardiovascular disease, their effect on T2DM patients with stabilized AMI remains unknown. Therefore, this study was designed to investigate the effect of SGLT2 inhibitors on long-term cardiovascular and renal outcomes in T2DM patients after successful revascularization and stabilization of AMI.

## Methods

### Patient Population

**Figure 1:**
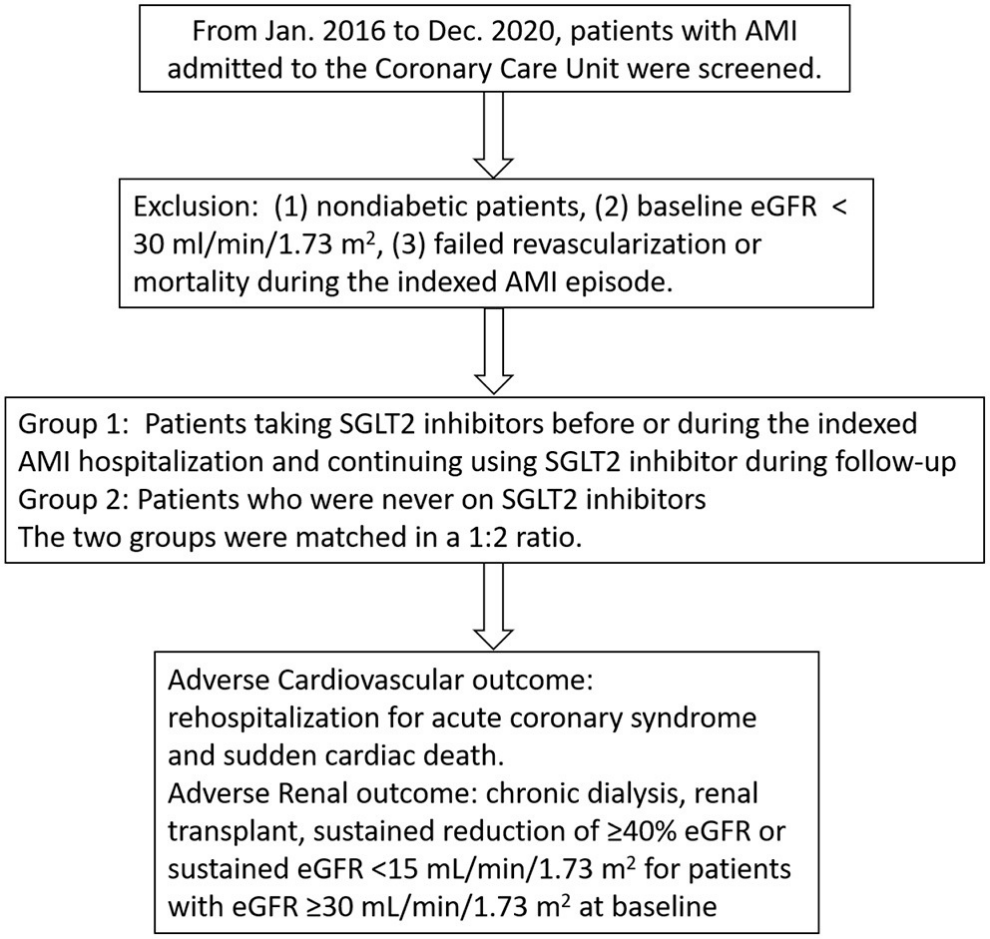
The flow diagram of this study.

The present study was approved by the Institutional Review Board at Taipei Veterans General Hospital, Taipei, Taiwan (IRB no. 2022-01-033CC). The data used in this study were anonymized before analysis. From January 2016 to December 2020, a total of 1,268 patients admitted to the Coronary Care Unit due to AMI were retrospectively screened in this study. The exclusion criteria were as follows: (1) nondiabetic patients, (2) those with a baseline estimated glomerular filtration rate (eGFR) of <30 mL/min/1.73 m^2^, (3) those who experienced failed revascularization or mortality during the index AMI episode. The patients who had been taking SGLT2 inhibitors before or during the index AMI episode, and continued using SGLT2i during the clinics follow-up, were defined as group 1. The patients who had never used SGLT2 inhibitors were defined as group 2. As shown in Figure 1, the study subjects of groups 1 and 2 were matched in terms of age, sex, congestive heart failure (CHF), and chronic kidney disease (CKD) at a 1:2 ratio. The definition of AMI was based on the diagnostic criteria; AMI was diagnosed by a coronary angiogram and confirmed by two expert cardiologists (21). By definition, all of the enrolled patients received successful revascularization and were discharged with stable conditions.

### Data Collection

The demographic profiles, cormobidities, laboratory data, and medication profiles of the participants were collected by the review of medical records. After being discharged, all of the participants were scheduled to have a follow-up visit at the clinic 2 weeks later. The subsequent clinic visits were arranged at 1–3-month intervals, according to the discretion of the attending cardiologist. Data of the follow-up period were obtained by a medical record review and confirmed by a telephone interview. The composite of rehospitalization due to acute coronary syndrome (ACS) or sudden cardiac death was defined as an adverse cardiovascular outcome. Meanwhile, the composite of the initiation of chronic dialysis, renal transplant, eGFR decline of ≥40%, or eGFR of <15 mL/min/1.73 m^2^ was defined as an adverse renal outcome. Chronic dialysis was defined as dialysis treatment for more than 90 days (22).

### Statistical Methods

All analyses were performed using SPSS statistical software, version 24.0 (SPSS, Inc., Chicago, IL, USA). Continuous variables were reported as the mean and standard deviation. Categorical variables were reported as frequencies and percentages. The chi-squared test was used to analyze categorical variables. Kaplan–Meier survival curves with the log-rank test were used to compare the occurrence of outcomes. The Cox proportional regression model was used to calculate the hazard ratio (HR) for the occurrence of outcomes.

## Results

### Baseline Characteristics of the Study Subjects

This retrospective observational study included 198 AMI patients, among whom 66 patients were in group 1, and 132 patients were in group 2. The mean age of the patients was 66.1 ± 12.3 years old in group 1 and 67.7 ± 11.9 years old in group 2. The age, sex, comorbidities, and baseline eGFR were similar between the two groups. Notably, the percentage of patients with ST-elevation myocardial infarction, the thrombolysis in myocardial infarction (TIMI) risk score, and the volume of contrast medium used for revascularization were not significantly different between the two groups (Table 1). The above findings suggest that the two matched groups presented to the hospital with a similar severity of AMI. In group 1, the majority of SGLT2 inhibitor prescription was empagliflozin (78.8%), and 12(18.2%) patients have been using the SGLT2 inhibitors before the indexe AMI hospitalization, with the mean duration of 12.8 ± 9.2 months.

**Table 1:** Baseline characteristics of the study subjects.

	**Group 1** **(*n* = 66)**	**Group 2** **(*n* = 132)**	***P*-value**
Age, years	66.1 ± 12.3	67.7 ± 11.9	0.382
Male, *n* (%)	50 (75.8%)	95 (71.9%)	0.612
Dyslipidemia, *n* (%)	21 (31.8%)	40 (30.3%)	0.871
Diabetes mellitus, *n* (%)	66 (100.0%)	132 (100.0%)	-
Congestive heart failure, *n* (%)	5 (7.6%)	6 (4.5%)	0.511
Hypertension, *n* (%)	44 (66.7%)	97 (73.5%)	0.323
Coronary artery disease, *n* (%)	66 (100.0%)	132 (100.0%)	-
Chronic kidney disease, *n* (%)	5 (7.6%)	15 (11.4%)	0.464
Old stroke, *n* (%)	4 (6.1%)	11 (8.3%)	0.777
Atrial fibrillation, *n* (%)	7 (10.6%)	10 (7.6%)	0.591
SGLT2 inhibitor			
Empagliflozin, *n* (%)	52 (78.8%)	0 (0%)	-
Dapagliflozin, *n* (%)	12 (18.2%)	0 (0%)	-
Canagliflozin, *n* (%)	2 (3%)	0 (0%)	-
Insulin therapy, *n* (%)	8 (12.1%)	20 (15.2%)	0.668
Left ventricular ejection fraction, %	52.0 ± 12.8	52.3 ± 10.6	0.873
eGFR, mL/min/1.73 m^2^	72.1 ± 22.7	67.7 ± 18.6	0.172
ST elevation myocardial infarction, *n* (%)	30 (45.5%)	48 (36.4%)	0.222
TIMI risk score	3.8 ± 1.8	4.1 ± 1.5	0.282
Contrast medium during revasculization, mL	204.6 ± 97.9	217.1 ± 106.3	0.473
Adverse cardiovascular outcomes, *n* (%)	4 (6.1%)	29 (22.0%)	0.004
Rehospitalization for ACS, *n* (%)	3 (4.5%)	22 (16.7%)	-
Sudden cardiac death, *n* (%)	1 (1.5%)	7 (5.3%)	-
Adverse renal outcomes, *n* (%)	4 (6.1%)	17 (12.9%)	0.220

*ACS, Acute coronary syndrome; eGFR, estimated glomerular filtration rate; SGLT2, sodium glucose cotransporter 2; TIMI, thrombolysis in myocardial infarction*.

### Risk of Adverse Cardiovascular and Renal Outcomes

**Figure 2:**
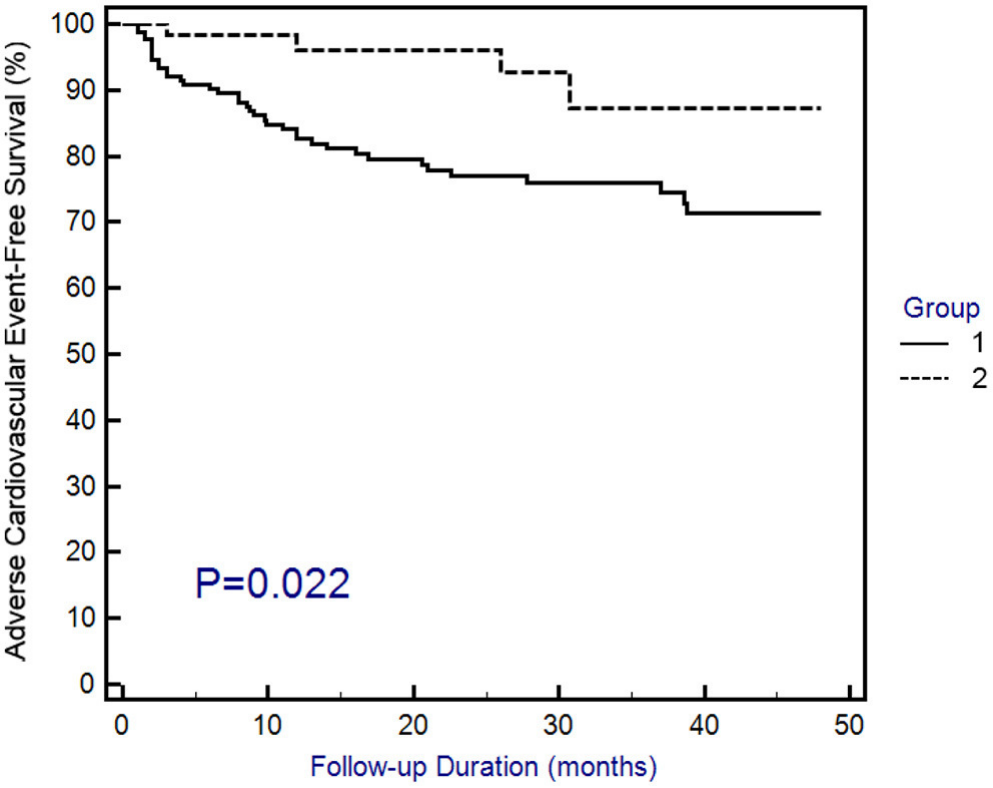
Kaplan–Meier curves of adverse cardiovascular outcomes. Adverse cardiovascular outcomes were defined as the composite of rehospitalization due to acute coronary syndrome or sudden cardiac death. Statistical analysis was performed using the log-rank test.

With a mean follow-up period of 23.5 ± 15.7 months (data not shown), 3 (4.5%) patients in group 1 and 22 (16.7%) patients in group 2 experienced rehospitalization due to ACS, while 1 (1.5%) patient in group 1 and 7 (5.3%) patients in group 2 experienced sudden cardiac death. Overall, the adverse cardiovascular outcomes occurred more frequently in group 2 (Table 1). To confirm the higher risk of adverse cardiovascular outcomes in group 2, Kaplan–Meier survival curves were constructed. The results demonstrated that the patients in group 1 had a longer adverse cardiovascular outcome-free survival than the patients in group 2 (Figure 2). The above findings suggest that post-AMI patients treated with SGLT2 inhibitors have a lower risk of adverse cardiovascular outcomes than those who are not taking this class of drug.

**Figure 3:**
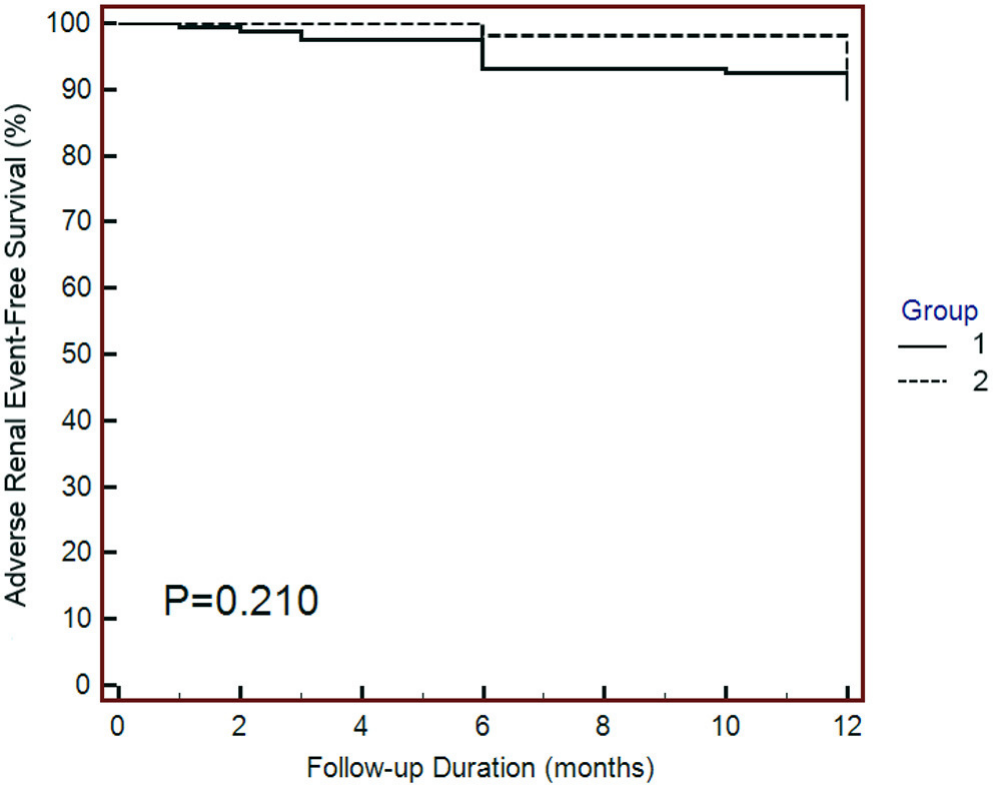
Kaplan–Meier curves of adverse renal outcomes. Adverse renal outcomes were defined as chronic dialysis, renal transplant, sustained reduction of ≥40% eGFR, or sustained eGFR <15 mL/min/1.73 m^2^ for patients with eGFR ≥30 mL/min/1.73 m^2^ at baseline. The adverse renal outcomes were similar between the two groups. Statistical analysis was performed using the log-rank test.

Regarding the adverse renal outcomes, there were 4 (6.1%) events in group 1 and 17 (12.9%) events in group 2; however, the difference was not significantly different between the two groups (Table 1). Similarly, the Kaplan–Meier survival curves showed that the adverse renal outcome-free survival was not significantly different between the two groups (Figure 3). The above findings suggest that SGLT2 inhibitors do not change the risk of adverse renal outcomes in post-AMI patients.

### Predictors of Rehospitalization for ACS and Sudden Cardiac Death

Compared with the patients without adverse cardiovascular outcomes, those who experienced adverse cardiovascular outcomes were older (72.3 ± 14.6 years old vs. 68.8 ± 11.5 years old, *P* = 0.045) and had a lower baseline eGFR (58.0 ± 20.8 vs. 69.3 ± 20.3, *P* = 0.004). Notably, the percentage of patients using SGLT2 inhibitors was significantly lower in the group of patients with an adverse cardiovascular event (12.1 vs. 37.6%, *P* = 0.004, Table 2).

**Table 2:** Comparison between patients with vs. without adverse cardiovascular events.

	**Patients with an adverse CV event**	**Patients without an adverse CV event**	
	**(*n* = 33)**	**(*n* = 165)**	***P*-value**
Age (years)	72.3 ± 14.6	66.8 ± 11.5	0.045
Male (*n*, %)	22 (66.7%)	132 (80%)	0.402
Dyslipidemia	11 (33.3%)	55 (33.3%)	0.838
Diabetes mellitus (*n*, %)	33 (100.0%)	165 (100.0%)	-
CHF (*n*, %)	4 (12.1%)	8 (4.8%)	0.097
Hypertension (*n*, %)	27 (81.8%)	127 (77.0%)	0.208
CAD (*n*, %)	33 (100.0%)	165 (100.0%)	-
CKD (*n*, %)	7 (21.2%)	20 (12.1%)	0.150
Old stroke (*n*, %)	1 (3%)	16 (9.7%)	0.482
Atrial fibrillation (*n*, %)	3 (9.1%)	16 (9.7%)	1.000
LVEF (%)	51.3 ± 9.8	52.1 ± 11.6	0.725
eGFR (mL/min/1.73 m^2^)	58.0 ± 20.8	69.3 ± 20.3	0.004
STEMI (%)	11 (33.3%)	70 (42.4%)	0.565
TIMI risk score (mean)	4.3 ± 1.4	4.0 ± 1.6	0.274
SGLT2 inhibitor	4 (12.1%)	62 (37.6%)	0.004

*ACS, acute coronary syndrome; CAD, coronary artery disease; CHF, congestive heart failure; CKD, chronic kidney disease; CV, cardiovascular; eGFR, estimated glomerular filtration rate; LVEF, left ventricular ejection fraction; SGLT2, sodium-glucose cotransporter 2; STEMI, ST-elevation myocardial infarction; TIMI, thrombolysis in myocardial infarction*.

To build the multivariate regression model, the candidate predictors were evaluated for their association with adverse cardiovascular outcomes using univariate Cox proportional regression analysis. The predictors with *P*-values of <0.05 were included in the multivariate Cox proportional regression model. Accordingly, age, baseline eGFR, and the use of SGLT2 inhibitors were included in the multivariate regression model. The results demonstrated that only the baseline eGFR (*P* = 0.008, 95% CI: 0.944–0.991) and the use of SGLT2 inhibitors (*P* = 0.039, 95% CI: 0.116–0.947) remained significantly associated with the risk of adverse cardiovascular outcomes (Table 3).

**Table 3:** Univariate and multivariate analyses of adverse cardiovascular outcomes.

	**Cox Univariate Analysis**		**Cox Multivariate Analysis**	
	***P*-value**	**95% CI**	***P*-value**	**95% CI**
Age (years)	0.008	1.010–1.038	0.272	0.985–1.055
Male (*n*, %)	0.454	0.368–1.564	-	-
Dyslipidemia	0.745	0.547–2.327		
Diabetes mellitus (*n*, %)	-	-	-	-
CHF (*n*, %)	0.145	0.764–6.195	-	-
Hypertension (*n*, %)	0.141	0.803–4.711	-	-
CAD (*n*, %)	-	-	-	-
CKD (*n*, %)	0.140	0.814–4.323		
Old stroke (*n*, %)	0.250	0.042–2.277	-	-
Atrial fibrillation (*n*, %)	0.924	0.323–3.472		
LVEF (%)	0.549	0.958–1.023		
eGFR (mL/min/1.73 m^2^)	0.003	0.953–0.990	0.008	0.944–0.991
STEMI (%)	0.272	0.210–1.551		
TIMI risk score (mean)	0.278	0.915–1.363	-	-
SGLT2 inhibitor use	0.030	0.110–0.895	0.039	0.116–0.947

*ACS, acute coronary syndrome; CAD, coronary artery disease; CHF, congestive heart failure; CKD, chronic kidney disease; eGFR, estimated glomerular filtration rate; LVEF, left ventricular ejection fraction; SGLT2, sodium-glucose cotransporter 2; STEMI, ST-elevation myocardial infarction; TIMI, thrombolysis in myocardial infarction*.

## Discussion

The main findings of this study were as follows: (1) In patients with T2DM and stabilized AMI, the use of SGLT2 inhibitors was associated with a lower risk of adverse cardiovascular outcomes. (2) Among diabetic patients with stabilized AMI, SGLT2 inhibitor use and a lower baseline renal function were both independent predictors of adverse cardiovascular outcomes. (3) The use of SGLT2 inhibitors was not significantly associated with adverse renal outcomes. These findings suggest that the use of SGLT2 inhibitors protects diabetic patients from AMI by reducing adverse cardiovascular events but does not change the risk of adverse renal outcomes in post-AMI patients.

Previous studies have demonstrated that in the early stage of AMI, SGLT2 inhibitor use reduces the myocardial infarct size through activation of signal transducer and activator of transcription 3 and downregulation of inflammatory responses in the infarcted myocardium (23). In addition, in diabetic mice, SGLT2 inhibitors reduce oxidative stress by decreasing the production of reactive oxygen species and the activity of nicotinamide-adenine dinucleotide phosphate (24, 25). Furthermore, SGLT2 inhibitors also have been shown to reduce oxidative stress through increasing endothelial nitric oxide synthase and nitric oxide formation in porcine endothelial cells (26).

Regarding atherosclerosis progression, previous studies have demonstrated that SGLT2 inhibitors suppress the transmigration of monocytes into the intraintimal space (24, 25, 27). Moreover, SGLT2 inhibitors also reduce the number of atheroma plaques as well as the size and the surface area of atherosclerotic lesions in animal models of diabetes and atherosclerosis (24, 25, 28–30). Additionally, SGLT2 inhibitors have been shown to stabilize atherosclerotic plaques by reducing the number of macrophages and the cholesterol crystal content in the atherosclerotic plaques (24, 29). The abovementioned studies suggest that SGLT2 inhibitors may treat the infarcted myocardium by restoring endothelial function, decreasing oxidative stress, reducing inflammation, and inhibiting the evolution of monocytes to macrophages to foam cells. Furthermore, SGLT2 inhibitors may prevent the progression of coronary atherosclerosis by reducing the plaque burden, changing the plaque composition, and increasing the plaque stability.

### Effects of SGLT2 Inhibitors on Post-infarcted Adverse Cardiac Remodeling

Following AMI, heart failure with adverse remodeling of left ventricle characterized by chamber dilatation and impaired cardiac function is the common outcome (31). In one recently published meta-analysis, including a total of 13 randomized controlled trials that evaluated the effects of SGLT2 inhibitors on cardiac remodeling in patients with T2DM and/or HF, SGLT2 inhibitors improved left ventricular (LV) ejection fraction, LV mass, LV mass index, LV end-systolic volume, LV end-systolic volume index, and E-wave deceleration time significantly (32). There were plausible effects of SGLT2 inhibitors on adversecardiac remodeling. First, myocardial ischemia could impair cardiomyocyte autophagy, which has been shown an essential mechanism that protects against adverse cardiac remodeling (33). Recent experimental studies have indicated that SGLT2 inhibitors might exert cardioprotective effects by stimulating autophagy (34). Second, cardiac mitochondrial dysfunction during ischaemia and reperfusion injury is a critical determinant of post-infarcted cardiac cell death, and is associated with future adverse cardiac remodeling (35). There have been several studies demonstrated that cardiac mitochondrial function could be improved by SGLT2 inhibitors (36–38). Taken together, reversed cardiac remodeling may be a mechanism responsible for the favorable clinical effects of SGLT2 inhibitor on patients with heart failure (39).

### Results of Previous Studies of SGLT2 Inhibitors to Treat Coronary Artery Disease

The meta-analysis of the four major CVOTs mentioned above showed a 12% reduction of the risk for MACE in the group taking SGLT2 inhibitors (HR, 0.88; 95%CI: 0.82–0.94) (40). In particular, there was a 17% reduction in sudden cardiac deaths and a 12% reduction in myocardial infarctions. In this meta-analysis, the authors also analyzed the effects of SGLT2 inhibitors in those with vs. without cardiovascular disease at baseline. The results showed that the risk reduction of myocardial infarction in the secondary prevention cohort was more significant than that in the primary prevention cohort (HR: 0.86; 95% CI: 0.80–0.93 for secondary prevention; and HR: 0.94; 95% CI: 0.82–1.07 for primary prevention). The other two meta-analysis studies also revealed risk reductions of 18% and 21%, respectively, for cardiovascular mortality in the SGLT2 inhibitor-treated group (41, 42). In accordance with these previous studies, our results showed that the patients taking SGLT2 inhibitors had fewer adverse cardiovascular outcomes, including rehospitalization for ACS and sudden cardiac death. Since the study population consisted of patients with stabilized AMI, our results demonstrate the protective effect of SGLT2 inhibitors on the secondary prevention of AMI, which is in line with a previous report (40).

### Results of Previous Studies of SGLT2 Inhibitors to Treat Chronic Kidney Disease

The meta-analysis of four CVOTs, including 38,723 participants with T2DM, demonstrated a risk reduction of 35% for end-stage renal disease in patients taking SGLT2 inhibitors (43). Nonetheless, another meta-analysis showed that while treatment with SGLT2 inhibitors reduced the risk of major renal outcomes by 46% in patients with macroalbuminuria and atherosclerotic cardiovascular disease, it had no significant effect on renal outcomes in the subgroup with eGFR <60 mL/min/1.73 m^2^ (HR: 0.74, 95% CI: 0.51–1.06) (44). In the current study, we did not observe a significant benefit of SGLT2 inhibitors on renal outcomes in diabetic patients with AMI. This finding could be explained by the small number of cases analyzed or the limited observation period.

In the past two decades, although the mortality due to AMI has improved (45, 46), the long-term cardiovascular mortality and post-AMI heart failure remain significant issues (45, 47). Thus, cardiac protection with early reperfusion to reduce the size of the myocardial infarct and the incidence of post-AMI cardiovascular adverse events are important topics to be studied. To the best of our knowledge, this is the first study showing that SGLT2 inhibitor use reduces the risk of adverse cardiovascular outcomes in T2DM patients with stabilized AMI.

This study has several limitations that must be addressed. First, due to the long period of enrollment, heterogeneity in treatment/stenting strategies may exist and confound the analysis. A second limitation is the single-center design and the relatively small number of included patients. A third limitation is that the physician skills regarding coronary revascularization were not controlled, which may also confound the analysis. Fourth, although a propensity score-adjusted analysis was employed to minimize the selection bias, remaining bias may still affect the analysis. Fifth, the retrospective nature of this study and the low number of events in each group limited further important subgroup analyses, such as an analysis based on the presence of absence of heart failure.

In conclusion, the findings of the present study might suggest that in T2DM patients with stabilized AMI, the use of SGLT2 inhibitors is associated with a lower risk of adverse cardiovascular outcomes, including rehospitalization for ACS and sudden cardiac death. Our study also demonstrates at least partly that the use of SGLT2 inhibitors could provide cardioprotection to T2DM patients with AMI. However, studies with a larger sample size are needed to verify these findings.

## Data Availability Statement

The original contributions presented in the study are included in the article/supplementary materials, further inquiries can be directed to the corresponding author.

## Ethics Statement

The studies involving human participants were reviewed and approved by Institutional Review Board, Taipei Veterans General Hospital. Written informed consent for participation was not required for this study in accordance with the national legislation and the institutional requirements.

## Author Contributions

T-YC and C-TLu analyzed the data and drafted the manuscript. H-LH and R-HC collected and analyzed the data. C-CC and C-TLiu drew the tables and figures. P-HH and S-JL designed the study and revised the manuscript. All authors contributed to the article and approved the submitted version.

## Funding

This study was supported, in part, by research grants from the Ministry of Science and Technology of Taiwan (MOST 104-2314-B-075-047), the Novel Bioengineering and Technological Approaches to Solve Two Major Health Problems in Taiwan sponsored by the Taiwan Ministry of Science and Technology Academic Excellence Program (MOST 108-2633-B-009-001), the Ministry of Health and Welfare (MOHW 106-TDU-B-211-113001), Taipei Veterans General Hospital (V105C-0207, V106C-045), and Wan Fang Hospital, Taipei Medical University (110-SWF-02). These funding agencies had no influence on the study design, data collection or analysis, decision to publish, or preparation of the manuscript.

## Conflict of Interest

The authors declare that the research was conducted in the absence of any commercial or financial relationships that could be construed as a potential conflict of interest.

## Publisher's Note

All claims expressed in this article are solely those of the authors and do not necessarily represent those of their affiliated organizations, or those of the publisher, the editors and the reviewers. Any product that may be evaluated in this article, or claim that may be made by its manufacturer, is not guaranteed or endorsed by the publisher.

## References

[1] Goldberg RB. Cardiovascular disease in diabetic patients. Med Clin North Am. (2000) 84:81–93, viii. 10.1016/S0025-7125(05)70208-X 10685129

[2] Simpfendorfer C. Efficacy of beta blockade, thrombolytic therapy, and coronary angioplasty in diabetic patients with coronary artery disease. Cleve Clin J Med. (1993) 60:145–9. 10.3949/ccjm.60.2.145 8095191

[3] Garcia MJ McNamara PM Gordon T Kannel WB. Morbidity and mortality in diabetics in the Framingham population. Sixteen year follow-up study. Diabetes. (1974) 23:105–11. 10.2337/diab.23.2.105 4359625

[4] Yudkin JS Oswald GA. Determinants of hospital admission and case fatality in diabetic patients with myocardial infarction. Diabetes Care. (1988) 11:351–8. 10.2337/diacare.11.4.351 3402292

[5] Behar S Boyko V Reicher-Reiss H Goldbourt U. Ten-year survival after acute myocardial infarction: comparison of patients with and without diabetes. SPRINT Study Group Secondary Prevention Reinfarction Israeli Nifedipine Trial. Am Heart J. (1997) 133:290–6. 10.1016/S0002-8703(97)70222-9 9060796

[6] Chun BY Dobson AJ Heller RF. The impact of diabetes on survival among patients with first myocardial infarction. Diabetes Care. (1997) 20:704–8. 10.2337/diacare.20.5.704 9135930

[7] Miettinen H Lehto S Salomaa V Mahonen M Niemela M Haffner SM. Impact of diabetes on mortality after the first myocardial infarction. The FINMONICA Myocardial Infarction Register Study Group. Diabetes Care. (1998) 21:69–75. 10.2337/diacare.21.1.69 9538972

[8] Franklin K Goldberg RJ Spencer F Klein W Budaj A Brieger D. Implications of diabetes in patients with acute coronary syndromes. The Global Registry of Acute Coronary Events. Arch Intern Med. (2004) 164:1457–63. 10.1001/archinte.164.13.1457 15249356

[9] Zhou M Liu J Hao Y Liu J Huo Y Smith SC Jr. Prevalence and in-hospital outcomes of diabetes among patients with acute coronary syndrome in China: findings from the improving care for cardiovascular disease in china-acute coronary syndrome project. Cardiovasc Diabetol. (2018) 17:147. 10.1186/s12933-018-0793-x 30482187 PMC6258152

[10] Jellinger PS Handelsman Y Rosenblit PD Bloomgarden ZT Fonseca VA Garber AJ. American Association of Clinical Endocrinologists and American College of Endocrinology Guidelines for Management of Dyslipidemia and Prevention of Cardiovascular Disease. Endocr Pract. (2017) 23:1–87. 10.4158/EP171764.GL 28437620

[11] Collet JP Thiele H Barbato E Barthelemy O Bauersachs J Bhatt DL. 2020 ESC Guidelines for the management of acute coronary syndromes in patients presenting without persistent ST-segment elevation. Eur Heart J. (2021) 42:1289–367. 10.1093/eurheartj/ehaa575 32860058

[12] Lam CSP Chandramouli C Ahooja V Verma S. SGLT-2 inhibitors in heart failure: current management, unmet needs, and therapeutic prospects. J Am Heart Assoc. (2019) 8:e013389. 10.1161/JAHA.119.013389 31607208 PMC6818035

[13] McMurray JJV Solomon SD Inzucchi SE Kober L Kosiborod MN Martinez FA. Dapagliflozin in Patients with Heart Failure and Reduced Ejection Fraction. N Engl J Med. (2019) 381:1995–2008. 10.1056/NEJMoa1911303 31535829

[14] Packer M Anker SD Butler J Filippatos G Pocock SJ Carson P. Cardiovascular and Renal Outcomes with Empagliflozin in Heart Failure. N Engl J Med. (2020) 383:1413–24. 10.1056/NEJMoa2022190 32865377

[15] Zinman B Wanner C Lachin JM Fitchett D Bluhmki E Hantel S. Empagliflozin, Cardiovascular Outcomes, and Mortality in Type 2 Diabetes. N Engl J Med. (2015) 373:2117–28. 10.1056/NEJMoa1504720 26378978

[16] Neal B Perkovic V Matthews DR Mahaffey KW Fulcher G Meininger G. Rationale, design and baseline characteristics of the CANagliflozin cardioVascular Assessment Study-Renal (CANVAS-R): A randomized, placebo-controlled trial. Diabetes Obes Metab. (2017) 19:387–93. 10.1111/dom.12829 28120497 PMC5348724

[17] Wiviott SD Raz I Bonaca MP Mosenzon O Kato ET Cahn A. Dapagliflozin and Cardiovascular Outcomes in Type 2 Diabetes. N Engl J Med. (2019) 380:347–57. 10.1056/NEJMoa1812389 30415602

[18] Cannon CP Pratley R Dagogo-Jack S Mancuso J Huyck S Masiukiewicz U. Cardiovascular Outcomes with Ertugliflozin in Type 2 Diabetes. N Engl J Med. (2020) 383:1425–35. 10.1056/NEJMoa2004967 32966714

[19] Zelniker TA Wiviott SD Raz I Im K Goodrich EL Bonaca MP. SGLT2 inhibitors for primary and secondary prevention of cardiovascular and renal outcomes in type 2 diabetes: a systematic review and meta-analysis of cardiovascular outcome trials. Lancet. (2019) 393:31–9. 10.1016/S0140-6736(18)32590-X 30424892

[20] Cosentino F Grant PJ Aboyans V Bailey CJ Ceriello A Delgado V. 2019 ESC Guidelines on diabetes, pre-diabetes, and cardiovascular diseases developed in collaboration with the EASD. Eur Heart J. (2020) 41:255–323. 10.1093/eurheartj/ehz486 31497854

[21] Thygesen K Alpert JS Jaffe AS Chaitman BR Bax JJ Morrow DA. Fourth Universal Definition of Myocardial Infarction (2018). Circulation. (2018) 138:e618–e51. 10.1161/CIR.0000000000000617 30571511

[22] Zannad F Ferreira JP Pocock SJ Zeller C Anker SD Butler J. Cardiac and Kidney Benefits of Empagliflozin in Heart Failure Across the Spectrum of Kidney Function: Insights From EMPEROR-Reduced. Circulation. (2021) 143:310–21. 10.1161/CIRCULATIONAHA.120.051685 33095032 PMC7834910

[23] Andreadou I Efentakis P Balafas E Togliatto G Davos CH Varela A. Empagliflozin limits myocardial infarction in vivo and cell death in vitro: role of STAT3, mitochondria, and redox aspects. Front Physiol. (2017) 8:1077. 10.3389/fphys.2017.01077 29311992 PMC5742117

[24] Leng W Ouyang X Lei X Wu M Chen L Wu Q. The SGLT-2 Inhibitor Dapagliflozin Has a Therapeutic Effect on Atherosclerosis in Diabetic ApoE(-/-) Mice. Mediators Inflamm. (2016) 2016:6305735. 10.1155/2016/6305735 28104929 PMC5220517

[25] Ganbaatar B Fukuda D Shinohara M Yagi S Kusunose K Yamada H. Empagliflozin ameliorates endothelial dysfunction and suppresses atherogenesis in diabetic apolipoprotein E-deficient mice. Eur J Pharmacol. (2020) 875:173040. 10.1016/j.ejphar.2020.173040 32114052

[26] Park SH Belcastro E Hasan H Matsushita K Marchandot B Abbas M. Angiotensin II-induced upregulation of SGLT1 and 2 contributes to human microparticle-stimulated endothelial senescence and dysfunction: protective effect of gliflozins. Cardiovasc Diabetol. (2021) 20:65. 10.1186/s12933-021-01252-3 33726768 PMC7967961

[27] Liu Y Xu J Wu M Xu B Kang L. Empagliflozin protects against atherosclerosis progression by modulating lipid profiles and sympathetic activity. Lipids Health Dis. (2021) 20:5. 10.1186/s12944-021-01430-y 33436015 PMC7802233

[28] Pennig J Scherrer P Gissler MC Anto-Michel N Hoppe N Funer L. Glucose lowering by SGLT2-inhibitor empagliflozin accelerates atherosclerosis regression in hyperglycemic STZ-diabetic mice. Sci Rep. (2019) 9:17937. 10.1038/s41598-019-54224-9 31784656 PMC6884628

[29] Terasaki M Hiromura M Mori Y Kohashi K Nagashima M Kushima H. Amelioration of Hyperglycemia with a Sodium-Glucose Cotransporter 2 Inhibitor Prevents Macrophage-Driven Atherosclerosis through Macrophage Foam Cell Formation Suppression in Type 1 and Type 2 Diabetic Mice. PLoS ONE. (2015) 10:e0143396. 10.1371/journal.pone.0143396 26606676 PMC4659635

[30] Han JH Oh TJ Lee G Maeng HJ Lee DH Kim KM. The beneficial effects of empagliflozin, an SGLT2 inhibitor, on atherosclerosis in ApoE (-/-) mice fed a western diet. Diabetologia. (2017) 60:364–76. 10.1007/s00125-016-4158-2 27866224

[31] Sutton MG Sharpe N. Left ventricular remodeling after myocardial infarction: pathophysiology and therapy. Circulation. (2000) 101:2981–8. 10.1161/01.CIR.101.25.2981 10869273

[32] Zhang N Wang Y Tse G Korantzopoulos P Letsas KP Zhang Q. Effect of sodium-glucose cotransporter-2 inhibitors on cardiac remodelling: a systematic review and meta-analysis. Eur J Prev Cardiol. (2022) 28:1961–73. 10.1093/eurjpc/zwab173 34792124

[33] Wu X He L Chen F He X Cai Y Zhang G. Impaired autophagy contributes to adverse cardiac remodeling in acute myocardial infarction. PLoS ONE. (2014) 9:e112891. 10.1371/journal.pone.0112891 25409294 PMC4237367

[34] Packer M. Autophagy stimulation and intracellular sodium reduction as mediators of the cardioprotective effect of sodium-glucose cotransporter 2 inhibitors. Eur J Heart Fail. (2020) 22:618–28. 10.1002/ejhf.1732 32037659

[35] Zhou B Tian R. Mitochondrial dysfunction in pathophysiology of heart failure. J Clin Invest. (2018) 128:3716–26. 10.1172/JCI120849 30124471 PMC6118589

[36] Santos-Gallego CG Requena-Ibanez JA San Antonio R Ishikawa K Watanabe S Picatoste B. Empagliflozin Ameliorates Adverse Left Ventricular Remodeling in Nondiabetic Heart Failure by Enhancing Myocardial Energetics. J Am Coll Cardiol. (2019) 73:1931–44. 10.1016/j.jacc.2019.01.056 30999996

[37] Verma S Rawat S Ho KL Wagg CS Zhang L Teoh H. Empagliflozin Increases Cardiac Energy Production in Diabetes: Novel Translational Insights Into the Heart Failure Benefits of SGLT2 Inhibitors. JACC Basic Transl Sci. (2018) 3:575–87. 10.1016/j.jacbts.2018.07.006 30456329 PMC6234616

[38] Croteau D Luptak I Chambers JM Hobai I Panagia M Pimentel DR. Effects of Sodium-Glucose Linked Transporter 2 Inhibition With Ertugliflozin on Mitochondrial Function, Energetics, and Metabolic Gene Expression in the Presence and Absence of Diabetes Mellitus in Mice. J Am Heart Assoc. (2021) 10:e019995. 10.1161/JAHA.120.019995 34169737 PMC8403324

[39] Salah HM Verma S Santos-Gallego CG Bhatt AS Vaduganathan M Khan MS. Sodium-Glucose Cotransporter 2 Inhibitors and Cardiac Remodeling. J Cardiovasc Transl Res. (2022). 10.1007/s12265-022-10220-5. [Epub ahead of print]. 35290593

[40] Arnott C Li Q Kang A Neuen BL Bompoint S Lam CSP. Sodium-Glucose Cotransporter 2 Inhibition for the Prevention of Cardiovascular Events in Patients With Type 2 Diabetes Mellitus: A Systematic Review and Meta-Analysis. J Am Heart Assoc. (2020) 9:e014908. 10.1161/JAHA.119.014908 31992158 PMC7033896

[41] Zheng SL Roddick AJ Aghar-Jaffar R Shun-Shin MJ Francis D Oliver N. Association Between Use of Sodium-Glucose Cotransporter 2 Inhibitors, Glucagon-like Peptide 1 Agonists, and Dipeptidyl Peptidase 4 Inhibitors With All-Cause Mortality in Patients With Type 2 Diabetes: A Systematic Review and Meta-analysis. JAMA. (2018) 319:1580–91. 10.1001/jama.2018.3024 29677303 PMC5933330

[42] Zhu J Yu X Zheng Y Li J Wang Y Lin Y. Association of glucose-lowering medications with cardiovascular outcomes: an umbrella review and evidence map. Lancet Diabetes Endocrinol. (2020) 8:192–205. 10.1016/S2213-8587(19)30422-X 32006518

[43] Neuen BL Young T Heerspink HJL Neal B Perkovic V Billot L. SGLT2 inhibitors for the prevention of kidney failure in patients with type 2 diabetes: a systematic review and meta-analysis. Lancet Diabetes Endocrinol. (2019) 7:845–54. 10.1016/S2213-8587(19)30256-6 31495651

[44] Li N Lv D Zhu X Wei P Gui Y Liu S. Effects of SGLT2 Inhibitors on Renal Outcomes in Patients With Chronic Kidney Disease: A Meta-Analysis. Front Med (Lausanne). (2021) 8:728089. 10.3389/fmed.2021.728089 34790672 PMC8591237

[45] Schmidt M Jacobsen JB Lash TL Botker HE Sorensen HT. 25 year trends in first time hospitalisation for acute myocardial infarction, subsequent short and long term mortality, and the prognostic impact of sex and comorbidity: a Danish nationwide cohort study. BMJ. (2012) 344:e356. 10.1136/bmj.e356 22279115 PMC3266429

[46] Szummer K Wallentin L Lindhagen L Alfredsson J Erlinge D Held C. Improved outcomes in patients with ST-elevation myocardial infarction during the last 20 years are related to implementation of evidence-based treatments: experiences from the SWEDEHEART registry 1995-2014. Eur Heart J. (2017) 38:3056–65. 10.1093/eurheartj/ehx515 29020314 PMC5837507

[47] Chen J Hsieh AF Dharmarajan K Masoudi FA Krumholz HM. National trends in heart failure hospitalization after acute myocardial infarction for Medicare beneficiaries: 1998-2010. Circulation. (2013) 128:2577–84. 10.1161/CIRCULATIONAHA.113.003668 24190958 PMC4415510

